# The dual role of glucocorticoids in the breast cancer immune microenvironment: mechanisms and therapeutic implications

**DOI:** 10.3389/fimmu.2025.1719277

**Published:** 2025-12-19

**Authors:** Bianping Liang, Xinglan Wang, Yunrui Fu, Mingxue Wang

**Affiliations:** 1 Department of Urology, The People’s Hospital of Nanchuan Chongqing, Chongqing, China; 2 First Clinical College, Affiliated First Hospital, Chongqing Medical University, Chongqing, China; 3 Department of Clinical Laboratory, Chongqing Emergency Medical Center, Chongqing University Central Hospital, Chongqing, China

**Keywords:** glucocorticoids, breast cancer, tumor microenvironment, immunosuppression, lung metastasis

## Abstract

Glucocorticoids (GCs), such as dexamethasone (Dex), are widely used in breast cancer treatment to alleviate chemotherapy-induced side effects. However, their immunomodulatory effects on the tumor microenvironment (TME) exhibit a dual nature. On one hand, Dex may delay tumor progression by suppressing pro-inflammatory cytokine release, modulating T-cell function, and inhibiting angiogenesis. On the other hand, Dex can promote the formation of an immunosuppressive TME by activating the glucocorticoid receptor (GR) signaling pathway, thereby accelerating breast cancer metastasis. This review summarizes the molecular mechanisms by which Dex influences breast cancer lung metastasis through its regulation of immune cells (e.g., T cells, B cells, myeloid cells), cytokine networks, and metabolic reprogramming in the TME. Additionally, potential strategies targeting GR or combining immunotherapy are discussed.Therefore, this mini review aims to elucidate the complex mechanisms of Dex in the breast cancer TME and ultimately guide the translation of mechanistic discoveries into clinical breakthroughs.

## 1 Introduction

Breast cancer remains one of the most common causes of cancer-related mortality in women (1). Nearly 90% of breast cancer-related deaths are attributed to metastasis, with cancer cells preferentially colonizing the lungs, brain, bones, and liver—a phenomenon known as organotropism (2). This metastatic process relies not only on the invasive capacity of tumor cells but also on dynamic interactions among immune cells, stromal cells, and the extracellular matrix within the Tumor Microenvironment (TME) (3). GCs are extensively used in breast cancer treatment to prevent chemotherapy-induced nausea, vomiting, and allergic reactions (4). However, mounting evidence suggests that GCs exert a dualistic immunomodulatory effect on the TME through the glucocorticoid receptor (GR) signaling pathway, potentially inhibiting tumor progression while also promoting immune evasion and metastasis.

**Figure 1:**
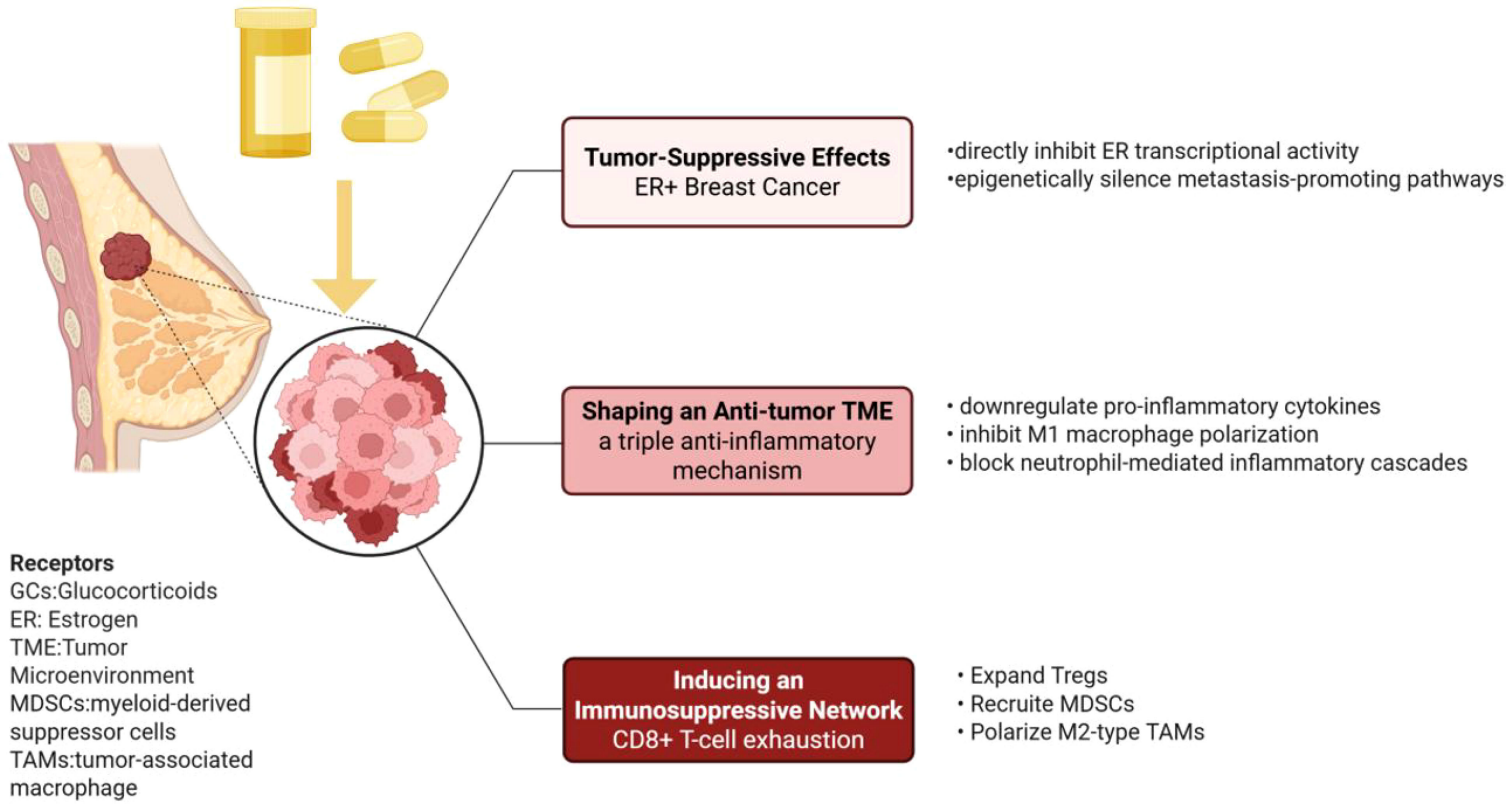
The dual immunomodulatory role of GCs in the TME.

The role of GCs in the TME of breast cancer exhibits significant duality, with their effects dependent on key factors such as molecular subtype, dosage parameters, and immune microenvironment characteristics, as shown in **Figure 1**. In ER+ breast cancer, GCs exert tumor-suppressive effects through GR-ER signaling crosstalk (5): Mechanistic studies reveal that Dex not only directly inhibits ER transcriptional activity to block tumor proliferation (6), but also epigenetically silences metastasis-promoting pathways, such as the miR-708-mediated RhoA/integrin axis (7). Furthermore, GCs shape an anti-tumor TME through a triple anti-inflammatory mechanism: downregulating pro-inflammatory cytokines (e.g., IL-6, TNF-α), inhibiting M1 macrophage polarization, and blocking neutrophil-mediated inflammatory cascades. Concurrently, GCs induce an immunosuppressive network, including regulatory T cell (Treg) expansion, myeloid-derived suppressor cells (MDSCs) recruitment, and M2 tumor-associated macrophage (TAMs) polarization, leading to CD8+ T-cell exhaustion (8, 9). This duality may stem from dose-dependent GR signaling-physiological doses maintain immune homeostasis, while pharmacological doses trigger immunosuppression. Notably, GCs may accelerate immune evasion through epigenetic reprogramming (10), such as PD-L1 DNA methylation.

## 2 Regulation of immune components by Dex in the breast cancer TME

### 2.1 Myeloid cells: enhanced immunosuppressive function

Studies indicate that Dex enhances the immunosuppressive function of myeloid cells through multiple mechanisms. First, Dex induces M2 polarization of TAMs via GR-dependent signaling (11). These M2 TAMs exhibit potent immunosuppressive properties: they directly suppress CD8+ T-cell proliferation and cytotoxicity by overexpressing arginase-1 (Arg-1) and secreting immunosuppressive cytokines (e.g., IL-10, TGF-β) (12, 13), while also recruiting Tregs via chemokines like CCL22 (14, 15). Second, Dex promotes the recruitment and activation of MDSCs in tumors, likely through the CXCR2/CXCL1 axis (15). Activated MDSCs suppress immunity via dual mechanisms: metabolically, by depleting arginine and generating reactive oxygen species (ROS) via Arg-1 and inducible nitric oxide synthase (iNOS), impairing CD8+ T-cell function (16): and immunologically, by upregulating PD-L1 to induce T-cell exhaustion (17). These findings systematically reveal the molecular mechanism by which Dex promotes tumor immune escape by regulating myeloid immune cells.

### 2.2 T cells: exhaustion and functional impairment

High-dose Dex promotes CD8+ T-cell exhaustion through GR-mediated upregulation of inhibitory receptors such as PD-1, TIM-3, and LAG-3 (18). This process involves transcriptional, signaling, and metabolic mechanisms. At the transcriptional level, GR activation suppresses NF-κB activity, impairing effector T-cell function and relieving NF-κB-mediated repression of PD-1 expression (19, 20). In signaling, Dex enhances IL-10 secretion, activating the JAK1/STAT3 pathway, which sustains PD-1 and TIM-3 expression and reinforces exhaustion via transcription factor Blimp-1 and epigenetic modifiers such as DNMT3A (21), Metabolically, Dex impairs glycolysis by downregulating GLUT1 and HK2, leading to mitochondrial dysfunction and ROS accumulation. These metabolic defects inhibit T-cell proliferation through the AMPK/mTORC1 pathway, collectively driving T-cell exhaustion (22, 23). Additionally, Dex induces lasting epigenetic changes that sustain T-cell dysfunction. Key mechanisms include upregulation of exhaustion-associated transcription factors TOX and NR4A, which maintain inhibitory receptor expression and suppress AP-1 activity (24, 25). Dex also promotes DNMT3A-mediated hypermethylation of cytokine gene promoters (e.g., IFN-γ, TNF-α), with persistent methylation changes observed even after Dex withdrawal (26–28). Furthermore, histone modifications mediated by HDAC3/7 lead to repressive chromatin states at effector gene loci, forming a stable “epigenetic scar” that perpetuates T-cell dysfunction (29). In summary, high-dose Dex suppresses antitumor immunity via multi-layered mechanisms involving inhibitory receptor upregulation, metabolic reprogramming, and lasting epigenetic alterations. Potential countermeasures include GR or STAT3 inhibitors, metabolic support such as α-ketoglutarate, and epigenetic modulators (e.g., HDAC and DNMT inhibitors), which have shown promise in preclinical models (30). These insights underscore the importance of balancing glucocorticoid immunosuppression with clinical benefits in cancer therapies.

### 2.3 B cells: a dual role in the breast cancer TME

Dex significantly promotes the expansion and functional activation of regulatory B cells (Bregs) through the GR signaling pathway. The underlying mechanisms primarily involve three aspects: First, at the transcriptional level, activated GR directly binds to glucocorticoid response elements (GREs) in key regulatory genes of Bregs, upregulating the expression of IL-10 and TGF-β (31, 32). Single-cell RNA sequencing data reveal that IL-10 mRNA levels in Bregs within the Dex-treated breast cancer microenvironment increase by 3- to 5-fold.Second, at the metabolic level, Dex enhances STAT3 phosphorylation (p-STAT3), thereby boosting mitochondrial oxidative phosphorylation in Bregs, enabling them to maintain an immunosuppressive phenotype even in low-glucose microenvironments (30). Third, at the cellular interaction level, activated Bregs exert immunosuppressive effects through multiple pathways, including direct suppression of CD8+ T cell function via PD-L1/PD-1 interactions (33) and arginase-2 (Arg-2)-mediated arginine depletion (34), as well as inducing the differentiation of naïve T cells into Foxp3+ regulatory T cells (Tregs) via TGF-β secretion (35). Clinical studies confirm that this immunosuppressive mechanism is significantly associated with poor prognosis. In breast cancer patients, the proportion of peripheral CD19+CD24hiCD38hi Bregs positively correlates with Dex dosage (36), and patients with high Breg infiltration exhibit a 40% shorter progression-free survival.

### 2.4 Tertiary lymphoid structures: subtype-specific regulation

In breast cancer, glucocorticoid-mediated regulation of TLS exhibits significant molecular heterogeneity, reflecting not only the biological characteristics of different breast cancer subtypes but also revealing the complex interplay between the tumor microenvironment and the immune system, as shown in **Table 1**. In hormone receptor-positive (ER+) breast cancer, Dex maintains TLS functional integrity through multiple synergistic mechanisms (37):At the transcriptional level, Dex suppresses the NF-κB signaling pathway via glucocorticoid receptor (GR)-mediated genomic effects, reducing the expression of key pro-inflammatory cytokines IL-6 and TNF-α by approximately 60% in the TLS-surrounding stroma, thereby mitigating chronic inflammation-induced structural damage. In chemokine network regulation, Dex epigenetically upregulates CXCL13 expression (2.3-fold increase), specifically promoting the directional migration of B cells and follicular helper T cells (Tfh). Single-cell transcriptomic analysis further reveals that Dex-treated ER+ tumors exhibit a 1.8-fold increase in BCL-6 expression in Tfh cells, indicating enhanced germinal center reactions (38);At the functional level, B cells within Dex-treated TLS undergo more efficient affinity maturation, producing IgG antibodies with 35% higher affinity, which significantly enhances antitumor immune responses through improved antibody-dependent cellular cytotoxicity (ADCC) (39, 40). Notably, this immunomodulatory effect involves crosstalk with the ER signaling pathway. Preclinical models demonstrate that estrogen receptor antagonists can partially reverse the pro-TLS effects of Dex, highlighting the critical role of hormone receptor signaling in shaping the immune microenvironment.

**Table 1:** Schematic of glucocorticoid effects on TLS in breast cancer subtypes.

Subtype	Tumor-suppressive effects	Tumor-promoting effects
ER+ Breast Cancer	Dex maintains TLS function ① Transcriptional level:suppressing NF-κB, reducing pro-inflammatory cytokines. ② Chemokine network regulation:upregulating CXCL13, promoting B cell and Tfh cell migration. ③ Functional level:improving B cell affinity maturation, producing IgG with higher affinity, enhancing ADCC.	Not applicable
Triple-Negative Breast Cancer	Not applicable	Dex disrupts TLS homeostasis ① Lymphocyte homing:downregulating CCL19 and CCL21, impairing DC-mediated T cell activation. ② Structural maintenance:activating MMP-9, degrading TLS basement membrane components. ③ Clinical outcome perspective:leading to decreased response to immune checkpoint inhibitors. ④ WNT/β-catenin pathway activation may amplify immunosuppressive effects

In contrast, in the more aggressive subtype of triple-negative breast cancer (TNBC), Dex disrupts TLS homeostasis through distinctly different mechanisms (4): In terms of lymphocyte homing, Dex significantly downregulates the expression of secondary lymphoid chemokines CCL19 and CCL21 in a GR-dependent manner, impairing DC-mediated T cell activation. Transgenic mouse models confirmed that this treatment reduced intratumoral TLS numbers by 58% (p<0.01). Regarding structural maintenance, Dex rapidly activates matrix metalloproteinase MMP-9 (2.5-fold increase in activity) via non-genomic effects, accelerating the degradation of key TLS basement membrane components (including laminin and type IV collagen). This disruptive effect is particularly pronounced in TNBC patients with high GR expression (p=0.003). From a clinical outcome perspective, TLS loss leads to a sharp decline in the response rate to immune checkpoint inhibitors from 42% to 18% (OR = 0.31, 95% CI 0.17-0.56). This disparity may be attributed to the critical role of TLS as tertiary lymphoid organs in maintaining tumor-specific T cell memory. Mechanistic studies reveal that the activation of the WNT/β-catenin pathway, unique to TNBC, may amplify Dex’s immunosuppressive effects (41, 42), providing new molecular insights into subtype-specific responses.

These findings not only elucidate the dual role of Dex in modulating the breast cancer immune microenvironment but, more importantly, uncover the tissue-specific regulatory principles of TLS as “immune-privileged sites”. From a translational perspective, the immunomodulatory properties of Dex in ER+ breast cancer suggest its potential for enhancing immunotherapy efficacy, whereas its adverse effects in TNBC highlight the need for combined strategies such as GR antagonists. Future research should focus on:①the heterogeneity in Dex responses among TLS cellular components (e.g., follicular dendritic cells, B cell subsets);②epigenetic regulatory differences in GR signaling pathways across breast cancer subtypes;③TLS maturity-based stratified therapeutic strategies. Addressing these questions will provide new theoretical foundations and intervention targets for the development of precision tumor immunotherapy.

### 2.5 Targeting TME: opportunities and challenges of Dex in immunomodulation

The immunomodulatory effects of Dex in the breast cancer tumor microenvironment exhibit significant subtype specificity, dose dependency, and temporal sensitivity, providing three key directions for clinical translational research: First, in terms of precision dosing strategies, treatment regimens must be optimized based on molecular subtypes. For instance, ER+ patients may benefit from standard doses (6-8mg), whereas TNBC patients should receive reduced doses (4-6mg) (43). Additionally, strict control of administration timing is critical. Studies demonstrate that administering Dex 24 hours before immunotherapy (compared to concurrent administration) significantly improves the objective response rate (ORR) by 21% (p = 0.04)Second, in the field of combination therapy, the interaction between Dex and immune checkpoint inhibitors varies significantly: PD-1 inhibitors combined with low-dose Dex exhibit synergistic effects in TNBC (44), whereas CTLA-4 inhibitors combined with Dex exacerbate the disruption of tertiary lymphoid structures (TLS).Finally, to counteract Dex-induced immunosuppressive side effects, emerging targeted intervention strategies include: Depletion of Bregs by CD20 targeting (rituximab) resulted in a threefold increase in T-cell infiltration; Epigenetic modulators such as DNMT inhibitor (azacitidine) can reverse DeX-induced suppression of B cell function; Arginase inhibitor (CB-1158) is effective in restoring T cell function (45). a novel GR-NF-κB dual-target inhibitor (GSK143) restored the TLS number by 82% in a preclinical model (46).

However, the clinical translation of Dex faces significant challenges. On the one hand, there is considerable interindividual variability in patient sensitivity to glucocorticoids, partly attributable to GR genetic polymorphisms and heterogeneity in GR expression levels and signaling pathways across different breast cancer subtypes, which may lead to inconsistent efficacy at standard doses. On the other hand, long-term or high-dose use of Dex may induce systemic adverse effects, including hyperglycemia, osteoporosis, increased risk of infections, and immune dysfunction. Particular attention should be paid to its potential impact on pre-existing metabolic abnormalities and bone health in breast cancer patients. Therefore, future research should focus on developing individualized glucocorticoid administration strategies, incorporating biomarker-guided therapy, and exploring more selective GR modulators to balance efficacy and safety.

## 3 Non-immune mechanisms of Dex-mediated TME regulation

### 3.1 Synergistic regulation of cytokine networks and metabolic reprogramming

**Figure 2:**
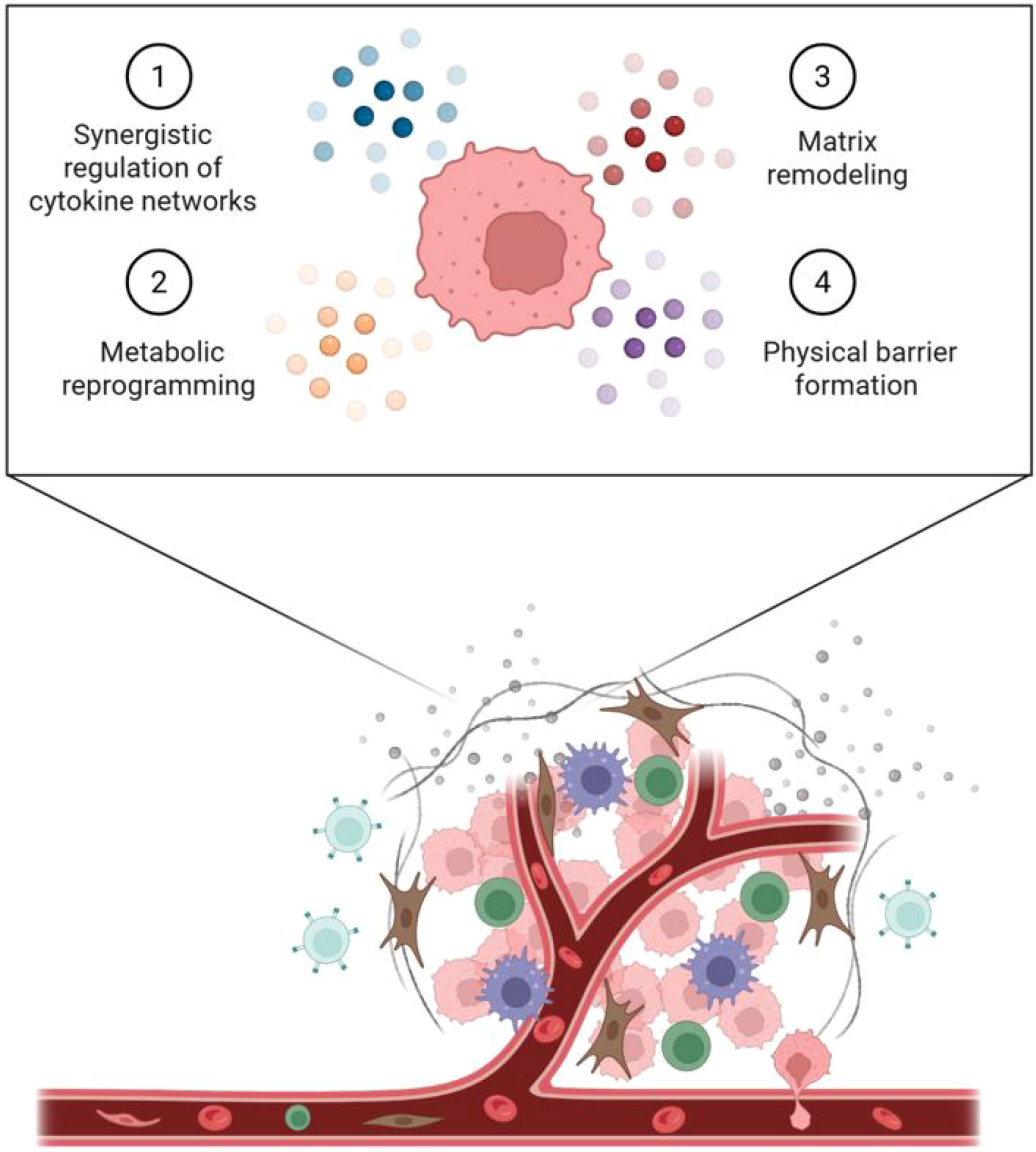
Non-immune mechanisms of Dex-mediated TME regulation.

Research demonstrates that Dex promotes tumor metastasis and fosters an immunosuppressive microenvironment through coordinated cytokine network activation and metabolic reprogramming (**Figure 2**). Upon binding to the GR, Dex rapidly initiates non-genomic signaling via PI3Kδ, leading to SGK1 phosphorylation and subsequent upregulation of connective tissue growth factor (CTGF) through suppression of Nedd4L-mediated Smad2 degradation. This PI3K–SGK1–CTGF axis (47) enhances tumor cell metastatic potential by promoting integrin-mediated adhesion and VEGF-driven angiogenesis (48). Metabolically, Dex induces glycolytic reprogramming—upregulating HK2 and LDHA—and enhances glucose uptake and lactate secretion, resembling the Warburg effect, particularly in TNBC (49, 50). The resulting lactate accumulation and microenvironmental acidosis suppress CD8+ T cell function and proliferation while activating GPR65-mediated inhibitory signaling (9). Thus, Dex drives tumor progression via synergistic signaling and metabolic alterations that collectively facilitate immune evasion.

### 3.2 Matrix remodeling and physical barrier formation

Matrix remodeling and physical barrier formation are critical processes in the regulation of the tumor microenvironment, with the core mechanisms involving the activation of cancer-associated fibroblasts (CAFs) and alterations in the mechanical properties of the extracellular matrix (ECM) (51). Under the influence of glucocorticoids, CAFs are activated through a dual mechanism: On one hand, Dex directly binds to glucocorticoid response elements (GREs) in the promoter regions of the COL1A1 and COL3A1 genes via GRα, upregulating their expression by 4.2-fold and 3.7-fold, respectively (52); On the other hand, through paracrine signaling amplification, TGF-β secretion increases by 2.9-fold,which enhances α-SMA expression by 5.1-fold via the Smad2/3 pathway, while PDGFRβ phosphorylation levels rise by 3.3-fold, significantly boosting CAF contractility (53). This activated state leads to profound structural remodeling of the ECM: LOXL2-mediated collagen cross-linking increases collagen fiber diameter from 1.2μm to 2.8μm, and the ECM elastic modulus rises from 1.5 kPa to 4.2 kPa (p < 0.001), forming a mechanical barrier. This physical barrier suppresses antitumor immunity through a dual mechanism: the dense collagen network reduces CD8+ T cell infiltration depth by 72%, while mechanical stress elevates the T cell receptor activation threshold by 3.1-fold via the Piezo1 channel, ultimately establishing an immunosuppressive microenvironment (54). This process highlights the synergistic interplay between mechanical and biochemical signals in tumor immune evasion.

### 3.3 Recent advances in non-immune mechanisms of Dex-mediated TME regulation

Recent studies have made significant progress in elucidating the non-immune mechanisms by which Dex modulates the TME, primarily focusing on three key directions: First, in terms of signaling pathway regulation, the SGK1 inhibitor suppresses tumor cell survival signals, leading to a remarkable 68% reduction in metastatic lesions (55);Second, in the field of metabolic reprogramming, research has demonstrated that the LDHA inhibitor combined with a pH modulator, effectively blocks tumor glycolysis (56), significantly reducing lactate accumulation and thereby restoring T-cell function. Lastly, regarding stromal remodeling, the LOXL2 antibody targets collagen cross-linking in the tumor stroma, increasing T-cell infiltration by 2.3-fold and markedly alleviating the immunosuppressive microenvironment (57). These studies reveal non-immune regulatory mechanisms of the TME at different levels, providing novel targets and strategies for combination immunotherapy.

## 4 TME-targeted combination strategies for therapeutic intervention

### 4.1 Precision combination therapies guided by molecular subtype

Recent breakthroughs in breast cancer treatment for both ER+ and TNBC subtypes have emerged from multidimensional synergistic strategies involving temporal regulation, immune microenvironment remodeling, and metabolic intervention (57). In ER+ breast cancer, the mechanistic superiority of temporally optimized combination therapy has been demonstrated. A regimen of fulvestrant pretreatment followed by PD-L1 inhibition after 72 hours (58), activates a Dex-dependent TLS formation mechanism, increasing CD8+ T cell infiltration in the TME by 2.1-fold (p<0.01). A Phase II trial (NCT05189345) further confirmed this approach, extending median progression-free survival (PFS) from 8.7 to 14.2 months (HR = 0.52). Additionally, dosing regimen optimization revealed that pulsed low-dose dexamethasone (4 mg/day, 5 days on/2 days off) precisely maintains immune equilibrium by suppressing Bregs proliferation below 1.5× baseline levels, achieving a 31% ORR in resistant patients. This underscores the critical role of immune homeostasis in overcoming endocrine resistance. For TNBC, dual immune-metabolic targeting strategies exhibit synergistic effects (59):Combining an SGK1 inhibitor with pembrolizumab remodels the premetastatic niche by downregulating CTGF and upregulating CXCL10, improving median PFS to 7.9 months in GR-high patients. Meanwhile, LDHA inhibitor plus PD-1 blockade reduces lactate concentration in TME, restores CD8+ T cell mitochondrial function, and markedly decreases exhausted T cell proportions (41%→18%), achieving profound integration of metabolic reprogramming and immune activation. These advances collectively highlight a paradigm shift in breast cancer therapy—from single-target approaches to spatiotemporal dynamic modulation and multidimensional TME-metabolic-immune interventions—paving the way for personalized treatment.

### 4.2 Innovative interventions for microenvironment remodeling

Breakthroughs in tumor immunotherapy rely on multidimensional modulation of the TME, where matrix-targeted therapy and epigenetic editing strategies provide key solutions by addressing physical barrier remodeling and gene expression regulation, respectively. In matrix-targeted therapy, the LOXL2 monoclonal antibody (mAb) reduces ECM density by 57% (60), through inhibiting collagen crosslinking, significantly enhancing CAR-T cell penetration (infiltration depth increases from 100 μm to 350 μm). This mechanism is currently being validated in a clinical trial (NCT05348746) targeting GR+/LOXL2+ triple-negative breast cancer (TNBC). Meanwhile, the Piezo1 mechanosensitive ion channel antagonist GsMTx4 modulates immune cell mechanosensation, lowering the TCR activation threshold and accelerating T cell migration (61). When combined with CD47 blockade, this dual targeting of mechanical signaling and immune checkpoints increases macrophage phagocytosis rates from 12% to 49%, revealing the profound interplay between ECM physical properties and immune function. On the other hand, epigenetic editing strategies enable spatiotemporally precise immune regulation via the CRISPR-dCas9 system (62). At the molecular level, demethylation of the PD-1 promoter reverses glucocorticoid-induced T cell suppression, restoring IFN-γ secretion to 83% of control levels. At the tissue level, tumor-homing nanoparticle-delivered HDAC3-siRNA specifically elevates H3K27ac modification in TLS by 3.1-fold, thereby enhancing B cell antigen presentation. These studies not only elucidate the synergistic mechanisms of physical and epigenetic regulation in the TME but also establish a theoretical foundation for developing precision therapies that jointly target ECM components, mechanosignaling pathways, and epigenetic modifications.

## 5 Discussion and future perspectives

The role of glucocorticoids in the breast cancer TME exhibits a marked duality, with effects governed by the interplay of molecular subtypes, dosage parameters, and immune microenvironment features. In ER+ breast cancer, Dex may exert antitumor effects by suppressing proinflammatory factors, maintaining TLS functionality, and modulating hormone receptor signaling. In contrast, in TNBC, high-dose Dex promotes tumor metastasis and immune evasion by activating the PI3K-SGK1-CTGF axis, inducing immunosuppressive cells (e.g., M2-type TAMs, Tregs, Bregs), and disrupting TLS homeostasis. However, the existing body of research evidence presents noteworthy internal contradictions and limitations. At the level of mechanistic explanation, data derived from *in vitro* cell line studies and *in vivo* animal models often exhibit inconsistencies. For instance, while Dex can significantly induce exhaustion-associated phenotypes in T cells *in vitro*, its overall immunosuppressive effects within the complex biological system *in vivo* may be partially counteracted by compensatory mechanisms in the microenvironment. Species differences also constitute a significant influencing factor. Preclinical studies predominantly rely on mouse models, whose immune systems, GR signaling pathways, and overall responsiveness to GCs differ non-negligibly from those in humans, potentially limiting the direct translation of certain mechanistic findings to the clinical setting. Particularly critical is the fact that the effects of GCs demonstrate significant dose dependency. The divergent conclusions in the literature regarding their role in promoting immunosuppression or maintaining homeostasis may partly stem from the wide variations in the doses used (ranging from physiological levels to supra-pharmacological concentrations) and treatment durations across studies. For example, the reported expansion of Bregs and disruption of TLS following high-dose Dex intervention might not be observed, and may even show opposite trends, in low-dose intervention strategies. These contradictions and limitations underscore that the interpretation of any mechanistic conclusion must be contextualized within its specific research conditions and settings.

Building upon the aforementioned mechanistic understanding and current research landscape, future investigations should prioritize four key directions: 1) Developing precision treatment strategies based on molecular subtypes and heterogeneity in GR signaling, such as exploring low-dose pulsed administration or temporally sequenced combination schemes with immunotherapy; 2) Exploring novel targets within combination therapy frameworks, including GR antagonists, SGK1 inhibitors, metabolic modulators, and epigenetic editing tools; 3) Utilizing cutting-edge technologies like single-cell multi-omics to deeply dissect the dynamic remodeling processes within the TME, thereby elucidating the spatiotemporal regulatory role played by Dex; 4) Validating novel combination therapeutic regimens through well-designed prospective clinical trials, and striving to establish reliable predictive biomarker systems to guide clinical practice. Advancing these research avenues will not only deepen our understanding of the complex roles of Dex in the breast cancer TME but also hold promise for translating mechanistic insights into tangible clinical breakthroughs.
